# Retraction and republication—Estimating dose-response relationships for vitamin D with coronary heart disease, stroke, and all-cause mortality: observational and Mendelian randomisation analyses

**DOI:** 10.1016/S2213-8587(23)00364-9

**Published:** 2024-01

**Authors:** The Editors of The Lancet Diabetes & Endocrinology

**Affiliations:** a The Lancet Diabetes & Endocrinology, London EC2Y 5AS, UK

On Oct 27, 2021, *The Lancet Diabetes & Endocrinology* published online the Article, “Estimating dose-response relationships for vitamin D with coronary heart disease, stroke, and all-cause mortality: observational and Mendelian randomisation analyses”, and the Article was published in print in the December, 2021, issue of the journal.^1^

On Nov 30, 2022, the editors of *The Lancet Diabetes & Endocrinology* were alerted by the authors and readers to concerns regarding the validity of the statistical methods used and, consequently, the results of the Mendelian randomisation analysis. On Dec 15, 2022, we published an exchange of letters where these concerns were raised, and the authors replied by describing their new statistical analysis and their new results.^2^, ^3^, ^4^ To further alert readers of the new analysis and the change to the original results, we published an Expression of Concern on July 13, 2023, and in view of the extent of changes necessary, we determined that the previous version of the Article should be retracted with a corrected version republished after re-review.^5^

Today we retract the previous version along with the accompanying comment by Guillaume Butler-Laporte and J Brent Richards^6^ and republish the corrected version of the Article^7^ and appendix which states that the authors' stratified Mendelian randomisation analyses do not support a causal relationship for 25(OH)D concentrations with either cardiovascular or mortality outcomes for individuals at all levels of 25(OH)D. The findings for the observational analysis remain the same. The updated version of the Article includes an appendix file annotating all changes made to the paper.
